# Supplementary material for: A new genus of sinogaleaspids (Galeaspida, stem-Gnathostomata) from the Silurian Period in Jiangxi, China
Source: PeerJ. 2020 May 15;8:e9008. doi: 10.7717/peerj.9008 (PMC7233274; doi:10.7717/peerj.9008)
Supplement: File S2 [file peerj-08-9008-s002.docx]

**Description of characters**

**[1] Median dorsal opening: (0) absent; (1) present.**

**[2] Festooned pattern of sensory canals on dorsal surface of headshield: (0) absent; (1) present.**

**[3] Aspidine tubercles: (0) absent; (1) present.**

**[4] Shape of headshield: (0) trapezoid-like; (1) triangular-like; (2) oval-like; (3) armet-like; (4) fork-like.**

**[5] Margin of headshield: (0) serrated; (1) smooth.**

**[6] Shape of median dorsal opening: (0) transverse slit-like; (1) oval-like; (2) slender longitudinal oval.**

**[7] Longitudinal oval dorsal opening: (0) not slit-like (length/width<6); (1) slit-like (length/width>6).**

Ratio between maximum length and maximum width of longitudinal oval median dorsal opening is a continuous quantitative data in Eugaleaspiformes. In order to integrate it into the discrete qualitative data of common phylogenetic analysis, it needs to be discretely processed. The most common method for performing discrete processing is to define an interval. When a continuous quantity data is in a certain interval, it corresponds to the corresponding feature state. For a more objective discrimination between states of character [7], we conducted a cluster analysis.

We measured 14 species possessing complete longitudinal oval dorsal opening in Eugaleaspiformes. Measured data see Table 1. The r ratio (r=a/b, Fig. 1A) were imported into SPSS v. 17.0. for Q- mode cluster analysis.

Table 1. Measured data of median dorsal opening in 14 Eugaleaspiformes taxa. a= maximum length of median dorsal opening; b= maximum width of median dorsal opening; r= ratio between a and b.

| Taxa | a | b | r=a/b |
| --- | --- | --- | --- |
| *Meishanaspis* | 9.0 | 2.5 | 3.7 |
| *Shuyu* | 3.0 | 1.5 | 2.0 |
| *Anjiaspis* | 6.3 | 3.5 | 1.8 |
| *Sinogaleaspis* | 4.0 | 0.9 | 4.4 |
| *Rumporostralis* | 6.5 | 1.3 | 5.0 |
| *Nochelaspis* | 22.3 | 3.2 | 7.0 |
| *Yunnanogaleaspis* | 15.7 | 3.2 | 4.9 |
| *Tridensaspis* | 14.0 | 2.2 | 6.4 |
| *Ptetogonaspis* | 10.4 | 1.8 | 5.8 |
| *Dunyu longiforus* | 29.3 | 3.1 | 9.5 |
| *Dunyu xiushanensis* | 35.8 | 5.3 | 6.8 |
| *Eugaleaspis changi* | 16.2 | 2.3 | 7.0 |
| *E. xujiachongensis* | 20.2 | 2.6 | 7.8 |
| *E. lianhuashanensis* | 35.0 | 4.0 | 8.8 |

**
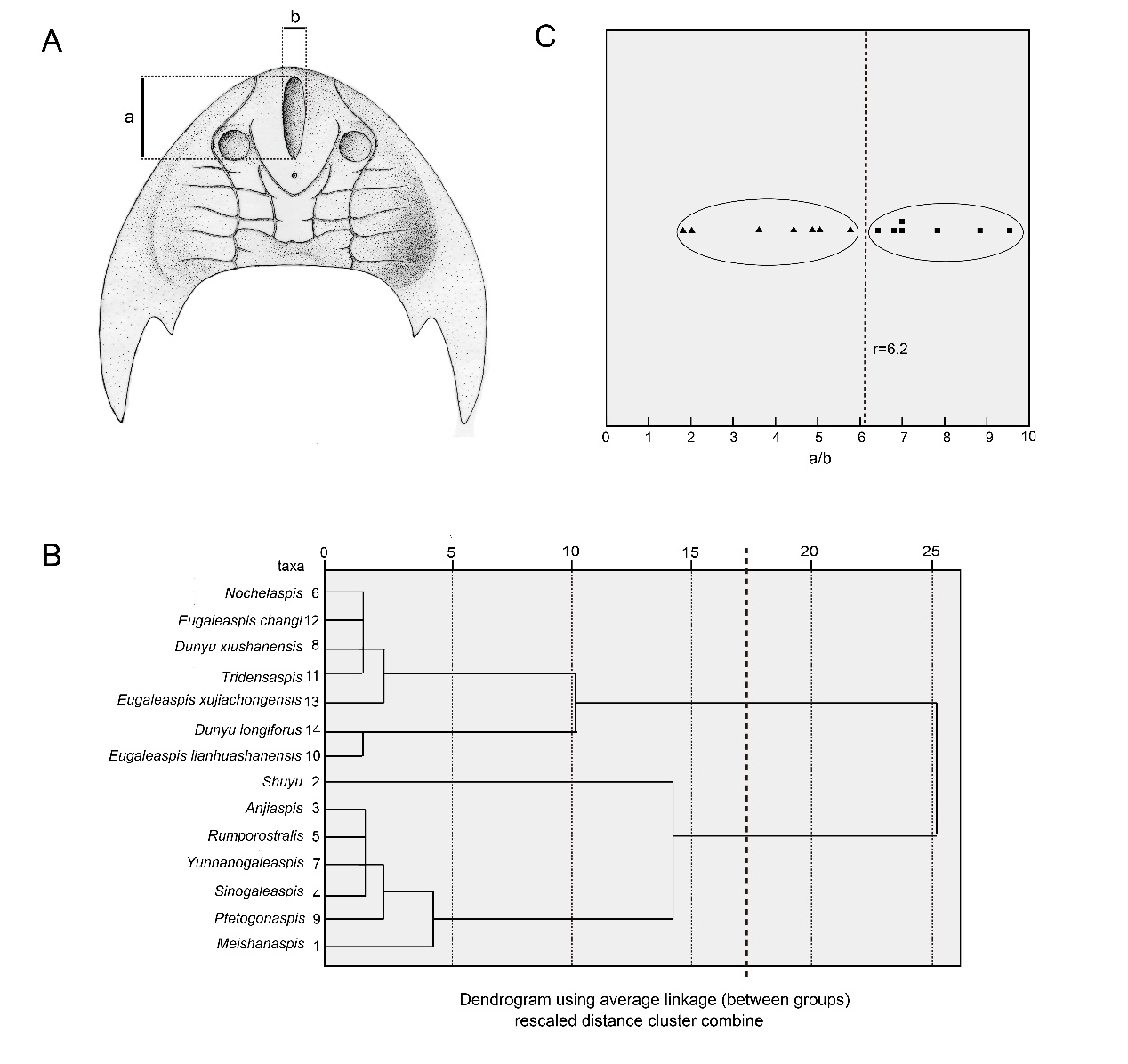
**

Fig.1 A, dorsal view of head-shield of Eugaleaspiformes showing the measurements (a, b). B, dendrogram generated by SPSS v17.0 using Q-mode cluster analysis. The dendrogram is rescaled into two clusters to meet the requirement of data discretion. C, dot plot of r using the new measured data; the data were cluster analysed and divided for character coding. Abbreviations: a, maximum length of median dorsal opening; b, maximum width of median dorsal opening.

We rescaled the resulting dendrogram into two clusters to meet the requirement of data discretion (Fig. 1B), which were constructed into a dot plot (Fig. 1C). Points of different shapes represent different clusters. We drew the lines to reflect the discrimination between two clusters, and to reformulate the character [7] as follows:

State of longitudinal oval medial dorsal opening, measured by the ratio r (length/width)

state 0. r<6；we define it as not slit-like.

state 1. r>6；we define it as slit-like.

**[8] Heart-shaped median dorsal opening: (0) absent; (1) present.**

**[9] Position of median dorsal opening: (0) not subterminal; (1) subterminal.**

**[10] Anterior end of median dorsal opening: (0) subterminal; (1) some distance behind rostral margin of shield; (2) terminal.**

**[11] Posterior end of median dorsal opening: (0) in front of or level with anterior margin of orbital opening;** **(1) between anterior margin of orbital opening and posterior margin of orbital opening; (2) posteriorly beyond posterior margin of orbital opening.**

**[12] Size of orbital openings: (0) large; (1) small.**

**[13] Position of orbital openings: (0) dorsal position and close to mid-line of headshield; (1) dorsal position and not close to mid-line of headshield; (2) lateral position.**

**[14] Cornual process: (0) absent; (1) present.**

**[15] Extending trajectory of cornual process: (0) projecting posterolaterally; (1) projecting laterally; (2) projecting backward.**

**[16] Portion of headshield behind cornual process: (0) short; (1) long.**

**[17] Position of cornual process base: (0) near posterior end of headshield; (1) away from posterior end of headshield.**

**[18] Spines on coner: (0) absent; (1) present.**

**[19] Inner cornual process: (0) present; (1) absent.**

**[20] Shape of inner cornual process: (0) broad leaf-shaped; (1) spine-shaped.**

**[21] Rostral margin of headshield: (0) without rostral angle; (1) with rostral angle.**

**[22] Rostral process: (0) absent; (1) present.**

**[23] Shape of rostral process: (0) broad; (1) slender.**

**[24] Spines or tubercles on margin of rostral process: (0) absent; (1) present.**

**[25] Fenestrae on dorsal surface of headshield: (0) absent; (1) present.**

**[26] Size of dorsal fenestra: (0) small; (1) large.**

**[27] Shape of dorsal fenestra: (0) slender oval-like; (1) broad bean-like.**

**[28] Position of dorsal fenestra: (0) lateral-dorsal position; (1) dorsal position.**

**[29] Position of dorsal fenestra relative to orbital opening: (0) orbital opening inside, fenestra outside; (1) orbital opening outside, fenestra inside.**

**[30] Median transverse canals (mtc): (0) two; (1) one; (2) more than two.**

The detailed pattern of sensory canals is the important diagnostic character for galeaspids. The homology of the sensory canal system between galeaspids and other agnathans was well analysed by Liu (1986). It was proposed that different types of the disposition of the sensory canal system in various agnathan groups were derived from a generel pattern that is composed of the longitudinal stems and the transverse branches linking them. It is mainly based on the analysis of Liu (1986) that the outgroup comparison is used here to determine the character polarization about the sensory canal system.

Among galeaspids, there exist three conditions as regard to the median transverse canal. In general, when the sensory canals were well preseved, there is only one median transverse canal, which was named as the dorsal commissure (dcm, Fig. 4) (Liu, 1986) or median dorsal commissure (Liu, 1975; Pan, 1992). In *Dayongaspis*, *Changxingaspis*, *Hanyangaspis* (Fig. 1A, C, 4A), there are two median transverse canals. In *Sinogaleaspis shankouensis* (Fig. 1D) (Pan and Wang, 1980) and *Anjiaspis* (Gai and Zhu, 2005) , there are more than three median transverse canals. No matter which canal in the latter two conditions is homologous to the dorsal commissure, there must be a dorsal commissure. By the outgroup comparsion, the presence of the dorsal commissure should be plesiomorphic.

In several genera, such as *Duyunolepis*, *Paraduyunaspis*, *Neoduyunaspis* (P’an and Wang, 1978a), *Huananaspis* (Liu, 1975), *Wumengshanaspis* (Wang and Lan, 1984), *Lungmenshanaspis* (P'an et al., 1975), *Qingmenaspis* (Pan and Wang, 1980), *Microhoplonaspis*, *Macrothyraspis*, *Sinoszechuanaspis* (Pan, 1992), the sensory canal systems were not preserved (the latter two genera retained the posterior supraorbital canal, the infraorbital canal, and lateral transverse canals), the canal system characters were coded as unavailable.

**[31] Short branches running from posterior supraorbital canal (soc_1_): (0) absent; (1) present.**

**[32] Lateral transverse canal: (0) short; (1) long.**

**[33] Branching end of lateral transverse canal: (0) absent; (1) present.**

**[34] Lateral transverse canals leaving from infraorbital canal: (0) present; (1) absent.**

**[35] The lateral transverse canals leaving from infraorbital canal before ltc_d_: (0) present; (1) absent.**

**[36] Fourth lateral transverse canal (ltc_4_): (0) present; (1) absent.**

**[37] Lateral transverse canals behind ltc_4_: (0) present; (1) absent.**

**[38] Anterior supraorbital canal (soc_1_): (0) absent; (1) present.**

**[39] Posterior supraorbital canal (soc_2_): (0) present; (1) absent.**

**[40] Posterior supraorbital canals (soc_2_) meet with infraorbital canal (ifc): (0) no; (1) yes.**

**[41] Posterior supraorbital canals (soc_2_): (0) funnel-shaped; (1) parallel; (2) V-shaped.**

**[42] Branching end of posterior supraorbital canals (soc_2_): (0) absent; (1) present.**

**[43] Medial dorsal canal (mdc): (0) present; (1) degenerated; (2) absent.**

**[44] Medial dorsal canal and posterior supraorbital canal: (0) unjointed; (1) contact.**

**[45] U-shaped medial dorsal canal: (0) absent; (1) present.**

**[46] Portion of headshield behind dorsal commissure proportionally: (0) long; (1) short.**

**[47] Postbranchial wall: (0) short; (1) long.**

**[48] Elongated branchial region: (0) absent; (1) present.**

**[49] Number of branchial fossae: (0) 5~7 pairs; (1) 9~17 pairs; (2) more than 20 pairs.**

**[50] Maximum width of headshield placed: (0) posteriorly; (1) medially.**

**[51] Width/length in oval-like headshield: (0) < 1; (1) >1.**

**[52] Nearly parallel lateral margins of headshield: (0) absent; (1) present.**

**[53] Broad and large middle dorsal spine of headshield: (0) absent; (1) present.**

**[1]- [53] from Gai et al. 2006**

**[54] Broad ventral rim: (0) absent; (1) present. From Gai et al. 2018**

**[55] The unclosed rostral margin: (0) absent; (1) present.**

**[56] The ornamentation of the head-shield: (0) star-shaped tubercles; (1) tiny granular tubercles; (2) coarse granular tubercles.**

**[57] Pineal organ: (0) on front of or level with posterior margin of orbital opening; (1)behind posterior margin of orbital opening.**

**[58] Pre-pineal region longer than the post-pineal region in mid-line of head-shield: (0) absent; (1) present.**

**[59]** **The preorbital commissure：(0) absent; (1) presen**

**Character 41 and character 45 of *Meishanensis* were recoding to “0”.**

**After re-observation of the specimen of *Meishanensis,* we found that posterior supraorbital canals is funnel-shaped and U-shaped medial dorsal canal is absent in *Meishanensis*.**
